# Functional differentiation and adaptive responses of absorptive and transport roots in alpine grassland plants under nitrogen and phosphorus addition

**DOI:** 10.3389/fpls.2025.1747072

**Published:** 2026-01-15

**Authors:** Jinke Du, Xueqi Li, Qiang Sun, Lu Yang, Ying Li, Shikui Dong

**Affiliations:** School of Grassland Science, Beijing Forestry University, Beijing, China

**Keywords:** alpine grasslands, nitrogen and phosphorus addition, plant functional traits, Qinghai-Tibetan Plateau, root economics spectrum

## Abstract

Fine root functional traits are critical for shaping plant strategies in below-ground resource acquisition and ecosystem functioning. However, previous studies have predominantly focused on fine roots as a whole or overlooked functional differentiation between root order, making it challenging to elucidate the response patterns and driving mechanisms of different root orders under nutrient addition. We conducted this study with controlled nitrogen and phosphorus addition experiments in alpine meadow and steppe ecosystems on the Qinghai-Tibetan Plateau (QTP), systematically analysing the response and differentiation characteristics of two fine root types—absorptive roots and transport roots—in key traits including root diameter (RD), specific root length (SRL), root tissue density (RTD), and root nitrogen concentration (RN). The results indicated that absorptive roots predominantly exhibited a ‘high SRL, low RTD’ absorptive trait combination, whilst transport roots displayed a ‘low SRL, high RTD’ conservative trait profile, these differentiation patterns remained consistent across all nitrogen and phosphorus addition treatments. This functional differentiation remains stable across different nitrogen and phosphorus treatments and grassland types, corroborating the framework of functional specialisation within root functional modules. Within the two-dimensional space, both absorptive and transport roots formed trade-off structures along the ‘SRL–RD’ and ‘RTD–RN’ axes, reflecting the coexistence of dual-axis economic spectra: ‘acquisition–conservation’ and ‘independence–cooperation’. Overall, the alpine grasslands plants on the QTP adapt to cold and nutrient–poor environments by balancing structural and metabolic traits, thereby supporting a strategy of synergistic trait trade-offs for environmental adaptation.

## 1 Introduction

Fine roots are vital organs for plants to absorb nutrients and water from the soil, playing a pivotal role in the utilization of belowground resources and the regulation of biogeochemical cycles in terrestrial ecosystems. Investigating fine-root functional traits is fundamental to understanding below-ground ecological processes and ecosystem-level material cycling, and for predicting ecosystem functional changes driven by vegetation shifts (McCormack et al., 2015). Root functional traits act as crucial intermediaries which link the plant adaptive strategies and ecosystem processes (Freschet et al., 2021) and encompass multiple hierarchical dimensions, including morphological, anatomical, and physiological traits. Moreover, these traits collectively determine plant efficiency in acquiring and allocating below-ground resources (Kong et al., 2014; McCormack et al., 2015) and profoundly influence carbon cycling and community assembly through root production and turnover (Freschet et al., 2021). Therefore, systematically investigating fine root functional traits and their variation patterns is of great scientific importance for elucidating plant adaptive strategies to environmental changes and maintaining ecosystem functional stability.

The root economics spectrum (RES) is recognized as the belowground analogue of the leaf economics spectrum (Wright et al., 2004). The conceptual foundation of the RES can be traced to early works linking leaf and root strategies (Ryser and Lambers, 1995; Westoby and Wright, 2006; Freschet et al., 2010), and was later extended to herbaceous species (Roumet et al., 2016) These studies demonstrated that root traits reflect trade-offs between resource acquisition and conservation, similar to leaves in the Leaf Economics Spectrum. McCormack et al. (2015) subsequently introduced the absorptive–transport root (AR–TR) framework, providing a hierarchical basis to interpret this variation within fine-root systems.Within this framework, root traits express the balance between nutrient uptake capacity and the metabolic costs of tissue construction and maintenance. Variation in root diameter (RD), specific root length (SRL), root tissue density (RTD), and root nitrogen concentration (RN) delineates a primary acquisition–conservation axis of the RES (Freschet et al., 2010; Bergmann et al., 2020). Thinner roots with high SRL and RN but low RTD represent an acquisitive strategy characterized by inexpensive construction and rapid resource turnover, whereas thicker roots with low SRL and RN but high RTD reflect a conservative strategy emphasizing structural stability and long tissue lifespan (Reich, 2014; Weemstra et al., 2016). Such differentiation illustrates how plants coordinate structural and metabolic investments to balance resource acquisition and conservation in varying environments.

Nevertheless, the classical RES framework primarily describes interspecific variation across species, whereas functional differentiation within the root system, particularly between absorptive and transport roots, remains insufficiently understood under nutrient enrichment. Roots with strong absorptive capacity tend to be thinner, while roots of different diameters jointly maintain the balance between resource acquisition and construction costs. However, recent studies reveal that relationships among fine root traits are not strictly linear. Constrained by root geometry and anatomy—such as the allometric scaling between cortex and stele (Kong et al., 2014; Zhang et al., 2024)—relationships among RD, SRL, root tissue density (RTD) and root nitrogen concentration (RN) exhibit pronounced nonlinearity and environmental dependency. These relationships more accurately describe the role of root traits in plant resource economics (Kong et al., 2019; De La Riva et al., 2021), and both environmental influences and phylogenetic histories substantially modify their forms (Bergmann et al., 2020), forming an additional ‘independence–cooperation’ axis of root traits. Accordingly, the functional space of fine roots has been described as two-dimensional, consisting of an acquisition–conservation axis and a nearly orthogonal independence–cooperation axis that reflects whether plants rely on intrinsic root construction or mycorrhizal fungal collaboration for nutrient uptake (Brundrett, 2009; Carmona et al., 2021). However, empirical evidence for these dimensions remains uneven across systems, and it is still unclear whether the functional differentiation within root systems—particularly between absorptive and transport roots—is stable or plastic under nutrient enrichment and across contrasting alpine grassland types. This study, therefore, aims to clarify whether absorptive roots (AR) and transport roots (TR) maintain distinct functional differentiation under nitrogen (N) and phosphorus (P) additions, and how nutrient addition and grassland type jointly shape this differentiation.

Traditionally, fine roots have been defined as the roots with diameters< 2 mm, and treated as a single functional, homogeneous pool (Jackson et al., 1997; Pritchard and Strand, 2008), effectively simplifying the entire root system into a single unified functional category. However, this approach has been recognized as insufficient to capture the complexity and functional diversity within fine root systems (Jackson et al., 1997; Pregitzer et al., 2002). Increasing evidences indicate that fine roots are highly branched and functionally heterogeneous: lower-order roots primarily perform nutrient and water absorption, whereas higher-order roots are more involved in transport and structural support (Jackson et al., 1997; Guo et al., 2008). Consequently, root functions are more closely associated with branching order than with diameter alone (Goebel et al., 2011; Mucha et al., 2020). Therefore, a hierarchical classification of fine roots, rather than a broad definition based solely on the< 2 mm threshold, is necessary to better understand differences in root function and resource allocation strategies (McCormack et al., 2015; Iversen et al., 2017). This also provides a crucial foundation for elucidating the RES and the ‘independence-cooperation’ dimension.

Qinghai-Tibetan Plateau (QTP), as a climate-sensitive region and an ecological security barrier, is subjected to long-term constraints imposed by low temperatures, aridity, and nutrient limitation. Its alpine grassland ecosystems exhibit strong sensitivity to nitrogen deposition, phosphorus limitation and climate change (Xia and Wan, 2008), making the region a natural laboratory for investigating below-ground plant response mechanisms under global change. Although previous studies have demonstrated significant effects of nitrogen and phosphorus addition on plant leaf traits and community structure, and have preliminarily explored their relationships with soil processes (Comas et al., 2014; Li et al., 2019), most work has still primarily focused on aboveground components. Systematic studies examining how fine root functional traits respond to nitrogen and phosphorus additions across hierarchical levels remain limited (Freschet et al., 2021). In particular, in alpine grassland ecosystems, it remains unclear whether the primary RES axis and the mycorrhizal cooperation dimension may decouple under nutrient enrichment, and which environmental or phylogenetic factors drive such responses (Brundrett, 2009; Kong et al., 2014).

To fill these knowledge gaps, we conducted a controlled experiment with six nitrogen and phosphorus addition treatments in two typical types of alpine grasslands on the QTP (Alpine Meadow and Alpine Steppe). We compared key traits of absorptive and transport roots and identified their main drivers. Absorptive and transport roots represent two functional modules within fine-root systems, responsible for resource uptake and transport, respectively (McCormack et al., 2015). This modularity is critical because shifts in the balance between these root types under nitrogen (N) and phosphorus (P) addition may alter plant strategies of construction investment and resource acquisition. Evaluating how nutrient enrichment affects AR and TR differentiation can therefore test whether fine-root economics conform to, or deviate from, the predictions of the root economics spectrum (RES) framework. This study aims to address the following scientific questions: (1) Do absorptive and transport roots in the alpine grasslands exhibit differential responses in key functional traits under nitrogen and phosphorus addition, individually or in combination? How these differences have been shaped? (2) Under the combined influences of nutrient addition and environmental conditions, do plant root systems in alpine grasslands exhibit a decoupling between the primary axis of the root economics spectrum (RES) and the mycorrhizal cooperation dimension? Addressing these questions allows us to test whether internal functional differentiation within fine-root systems remains stable or becomes plastic under nutrient enrichment. Absorptive and transport roots represent two complementary functional modules within plant root systems, responsible for nutrient uptake and resource transport, respectively. This modularity is ecologically important because it provides a mechanistic basis for linking individual trait variation to the overall functional balance of plants under changing environmental conditions. By examining how nutrient addition and habitat context shape AR–TR differentiation, this study provides empirical evidence for how fine-root trait coordination contributes to plant adaptation and the maintenance of belowground ecosystem stability on the Qinghai–Tibet Plateau.

## 2 Materials and methods

### 2.1 Study sites description

**Figure 1:**
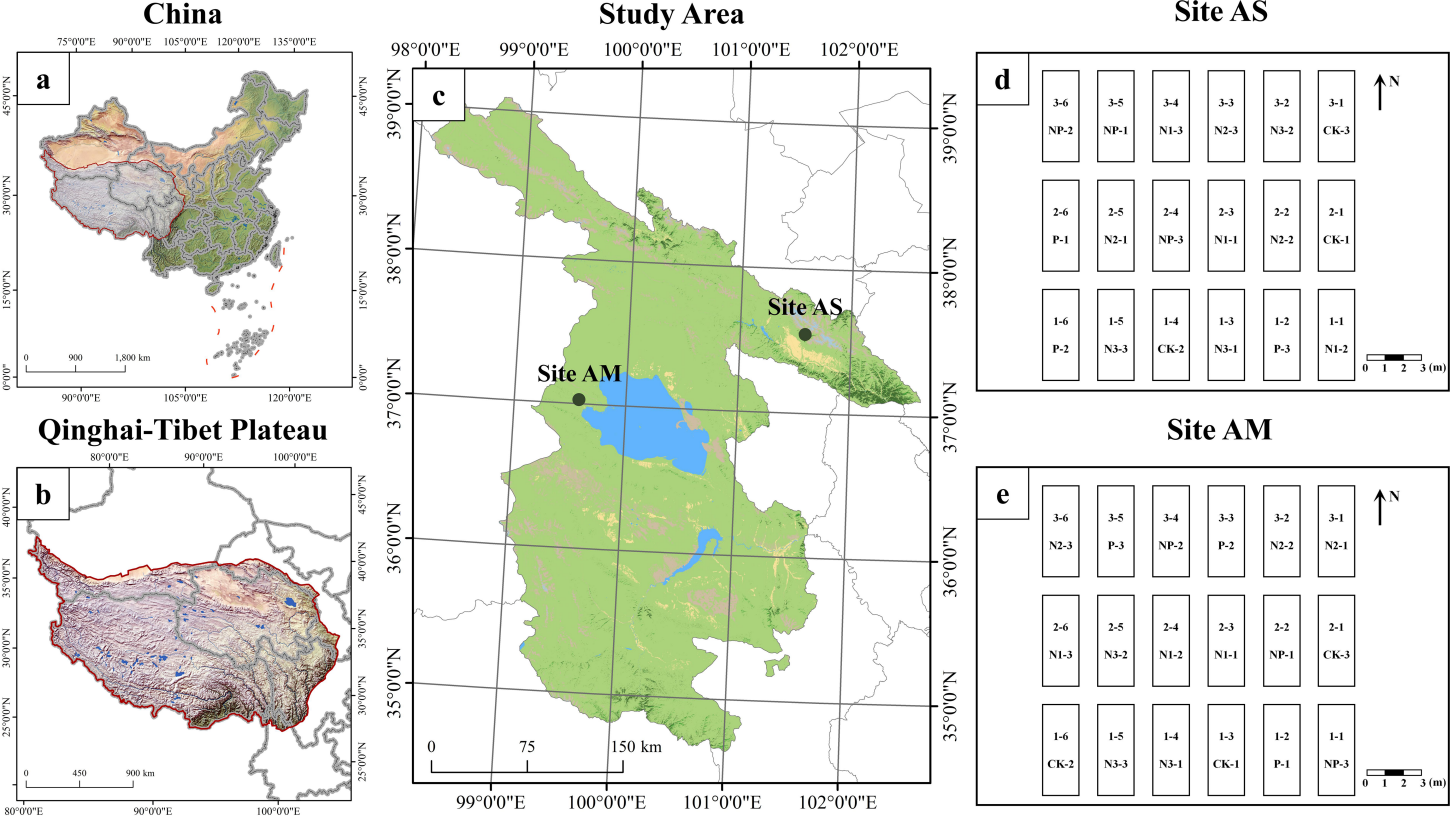
Location of the alpine grassland study area on the Qinghai-Tibet Plateau and layout of sample plots. Panel **(a)** shows the map of China; panel **(b)** illustrates the extent of the Qinghai-Tibet Plateau; panel **(c)** indicates the specific geographic location of the study area; panel **(d)** presents the layout of alpine grassland sample plots; and panel **(e)** presents the layout of alpine meadow sample plots.

This study was conducted in two representative alpine grassland ecosystems on the QTP, of China (**Figure 1**). The Alpine Meadow (AM) site is located at the Xihai Experimental Station in Haiyan County, Haibei Tibetan Autonomous Prefecture, Qinghai Province (36°93′N, 100°95′E; elevation 3100 m), situated within a plateau continental climate zone. The annual mean temperature is approximately 1.4°C, with annual precipitation ranging from 330 to 370 mm and potential annual evapotranspiration averaging around 1400 mm. Vegetation is dominated by typical alpine meadow species, including *Leymus secalinus*, *Agropyron cristatum*, *Stipa purpurea*, *Medicago ruthenica*, *Carex capillifolia*, *Carex alatauensis*, *Thalictrum petaloideum*, and *Potentilla multifida*. Alpine Steppe (AS) is situated at the Tiebujia Grassland Improvement Experimental Station in Gonghe County, Hainan Tibetan Autonomous Prefecture (37°02′N, 99°35′E; elevation 3270 m). It shares the same plateau continental climate zone, with a mean annual temperature of approximately 0°C, mean annual precipitation of 377 mm, and mean annual potential evapotranspiration of approximately 1484 mm. Vegetation is dominated by typical alpine steppe species, including *Leymus secalinus*, *Poa pratensis*, *Carex capillifolia*, *Thermopsis lupinoides*, and *Artemisia scoparia*, dominate the vegetation.

### 2.2 Experimental design and treatments

Since 2019, nitrogen addition experiments at varying levels have been conducted at the aforementioned experimental stations to simulate nitrogen deposition. Nitrogen (N) and phosphorus (P) were selected because N deposition has been rapidly increasing on the Qinghai-Tibet Plateau, while P availability remains relatively low and often limits plant growth and nutrient balance in alpine soils (Shen et al., 2022; Dong et al., 2023). Therefore, N addition was designed to represent different atmospheric deposition intensities, whereas a single P level was applied to relieve P limitation without causing nutrient imbalance or excessive precipitation in alkaline soils. Based on the region’s current and projected atmospheric nitrogen deposition levels (approximately 8 kg N·ha^−1^·yr^−1^) (Lü and Tian, 2007), six nutrient addition treatments were established: control (CK), low nitrogen addition (N1, 8 kg N·ha^−1^·yr^−1^, equivalent to local deposition levels), medium nitrogen addition (N2, 72 kg N·ha^−1^·yr^−1^, equivalent to 9 times local deposition levels), high nitrogen addition (N3, 216 kg N·ha^−1^·yr^−1^, equivalent to 27 times the local nitrogen deposition level), phosphorus addition (P, 35 kg P·ha^−1^·yr^−1^), and combined nitrogen-phosphorus addition (NP, 72 kg N·ha^−1^·yr^−1^ + 35 kg P·ha^−1^·yr^−1^) (Shen et al., 2022; Xiao et al., 2025). Each treatment comprised three replicates, totalling 18 plots (2 m×5 m each), arranged in a randomized block design. A 1-metre buffer zone was established between adjacent plots to minimize edge effects. Nitrogen was applied as ammonium nitrate (NH_4_NO_3_), and phosphorus as calcium dihydrogen phosphate (Ca(H_2_PO_4_)_2_). Fertilization was performed three times per year: May (early growth stage), July (peak growth stage), and September (late growth stage), to simulate the cumulative nature of atmospheric deposition and align with plant growth dynamics (**Supplementary Table S3**).

### 2.3 Root sampling and trait measurements

During the growing season (July-August) in 2024, representative areas (0.2 m×0.2 m) reflecting current plot characteristics were selected within each plot (Lu et al., 2024). Fine roots collected from each quadrat were first separated by species and then by root order before trait measurements, ensuring that all trait data represent species-level. Species identity of each root sample was determined by tracing root systems to their corresponding aboveground shoots within each quadrat, allowing accurate attribution of roots to plant species. All plants within the chosen area were carefully excavated using a sampling shovel. Soil adhering to the roots was gently removed by shaking, then washed off with deionized water. Living fine roots were identified based on colour, texture and elasticity. Following the root order classification framework proposed by McCormack et al. (2015) and King et al. (2021), fine roots were classified by order: the first and second order roots (orders 1-2) were defined as absorptive roots (AR), primarily responsible for water and nutrient uptake, while third to fifth order roots (orders 3-5) were defined as transport roots (TR), mainly involved in transport and structural support. These classified roots were then used for trait measurements. Each treatment included three replicate plots (n = 3 per treatment per site).

The classified fine roots were evenly spread in transparent root trays filled with deionized water and scanned using a digital scanner (Epson, Japan). The scanned images were analyzed using Win RHIZO root analysis software (Régent Instruments, Canada) to obtain root morphological traits including root length, average root diameter (RD; mm), and root volume. After scanning, root samples were oven-dried at 65 °C to constant weight for biomass determination, and subsequently used to calculate specific root length (SRL; m·g^−1^) and root tissue density (RTD; g·cm^−3^) according to Equations 1, 2, respectively. The dried samples were then ground into powder, and root nitrogen concentration (RN; g·kg^−1^) was quantified using an elemental analyzer.

(1) $$\begin{array}{c} \text{Specific Root Length }\left(\text{SRL}\;/\;\text{m}\cdot {\text{g}}^{-1}\right)=\text{Root}\;\text{Length}/\text{Dry}\;\text{Weight} \end{array}$$

(2) $$\begin{array}{c} \text{Root Tissue Density}\left(\text{RTD}\;/\;\text{g}\cdot \text{c}{\text{m}}^{-3}\right)=\text{Dry}\;\text{Weight}/\text{Root}\;\text{Volume} \end{array}$$

### 2.4 Data analysis

In total, six dominant species and six nutrient treatments (CK, N1, N2, N3, NP, and P) were included, resulting in 180 root samples (90 absorptive roots and 90 transport roots). Because not all species occurred in every treatment, the number of replicates per treatment varied slightly (**Supplementary Table S1**). Descriptive statistics, including mean, standard deviation, minimum, maximum, and coefficient of variation (CV), were calculated for each trait to characterize their distribution range. Normality and homogeneity of variance were first tested for all data. For traits meeting parametric assumptions, paired t-tests were used to assess differences between absorptive roots (AR) and transport roots (TR), and one-way analysis of variance (ANOVA) was applied to evaluate the effects of nitrogen and phosphorus treatments and grassland types. For traits that did not meet parametric assumptions, the Wilcoxon signed-rank test was employed. Statistical significance was set at α = 0.05. All trait data were analyzed at the species level, with intraspecific variation represented by individual replicates.

To explore the primary drivers of root-trait variation, a standardized difference index (diff) was calculated for each trait as according to Equation 3:

(3) $$\begin{array}{c} \text{diff}\;=\;\left({\text{X}}_{\text{TRR}}-{\text{X}}_{\text{ARR}}\right)/\left({\text{X}}_{\text{ARR}}+{\text{X}}_{\text{TRR}}\right) \end{array}$$

A linear mixed-effects model was then constructed with diff as the response variable according to Equation 4:

(4) $$\begin{array}{c} \text{diff}∼\text{Type}\times \text{Treatment}\times \text{Family}+(1|\text{Specie}\;1) \end{array}$$

Where Specie 1 was included as a random intercept to account for intraspecific variation, Type-III analysis of variance was used to assess the significance of fixed effects. Principal component analysis (PCA) was performed using the FactoMineR package in R to reveal multivariate relationships among root functional traits, including RD, SRL, RTD, and RN. Trait values were analyzed at the species × treatment × grassland-type level. Each treatment within a grassland type included three replicated plots (n = 3), covering 6 dominant species across both alpine meadow and alpine steppe, for a total of 216 species–trait observations. The corresponding sample sizes for each treatment and trait are summarized in **Supplementary Table S1**. To compare local trait patterns with the global root economics spectrum, global fine-root trait data were obtained from the GRooT database (Guerrero-Ramírez et al., 2021). Only herbaceous species with available data for RD, SRL, RTD, and RN were included. All trait variables were centered and scaled to unit variance (z-standardized) prior to analysis to ensure comparability across traits of different units. The global PCA was constructed using only the GRooT dataset, and the Qinghai–Tibet Plateau samples from this study were subsequently projected as supplementary scores to visualize their positions relative to the global root economic space.

All statistical analyses and visualizations were conducted in R version 4.5.1.

## 3 Results

### 3.1 Differences in root functional traits between absorptive roots and transport roots

**Figure 2:**
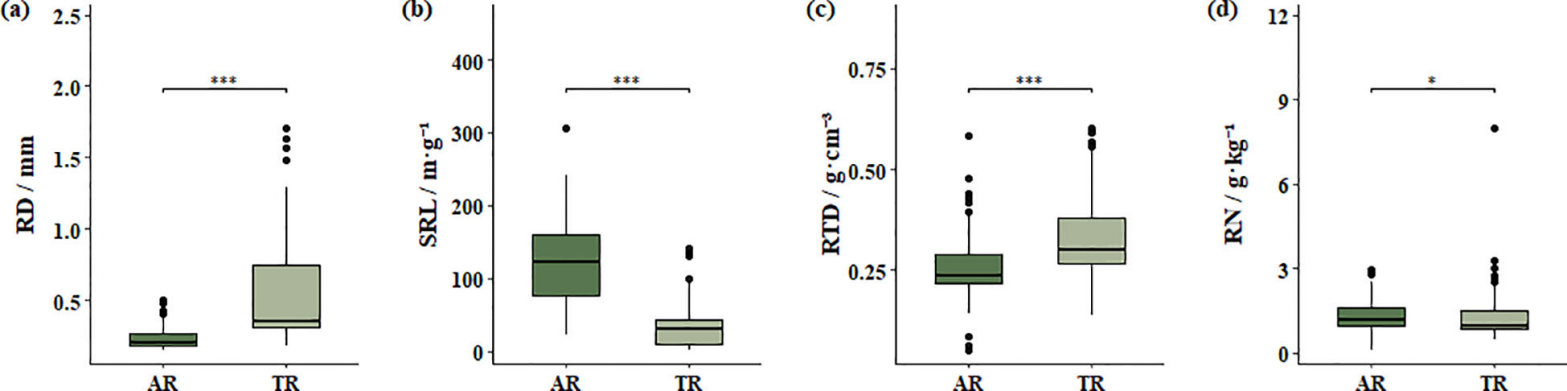
Comparison of functional traits between absorptive roots (AR) and transport roots (TR). **(a)** Root diameter (RD), **(b)** specific root length (SRL), **(c)** root tissue density (RTD), and **(d)** root nitrogen concentration (RN). * denotes significant differences between absorptive roots and transport roots (*P ≤ 0.05; ***P ≤ 0.001).

Our results showed significant differences between absorptive roots (AR) and transport roots (TR) for all measured root functional traits including root diameter (RD), specific root length (SRL), root tissue density (RTD), and root nitrogen concentration (RN) (**Supplementary Table S1**). Specifically, the RD of AR was 0.236 ± 0.080 mm, significantly smaller than that of TR (0.539 ± 0.391 mm; P< 0.001), indicating that AR was thinner and more favourable for rapid soil exploration and resource acquisition (**Figure 2a**). The RTD of AR was 0.259 ± 0.089 g·cm^−3^, significantly lower than that of TR (0.332 ± 0.107 g·cm^−3^; P< 0.001), suggesting that TR possessed stronger tissue structures supporting mechanical stability and resource transport (**Figure 2b**).

SRL of AR was 119.57 ± 56.09 m·g^−1^, significantly higher than that of TR (34.50 ± 30.65 m·g^−1^; P< 0.001), indicating a growth strategy optimized for efficient resource foraging at the distal portions of roots (**Figure 2c**). RN did not differ significantly between AR and TR (1.33 ± 0.55 g·kg^−1^ vs. 1.29 ± 0.96 g·kg^−1^; P > 0.05), although AR showed a slightly higher RN level consistent with their absorptive function (**Figure 2d**).

### 3.2 Root trait differences between absorptive roots and transport roots under nitrogen and phosphorus addition and across grassland types

**Figure 3:**
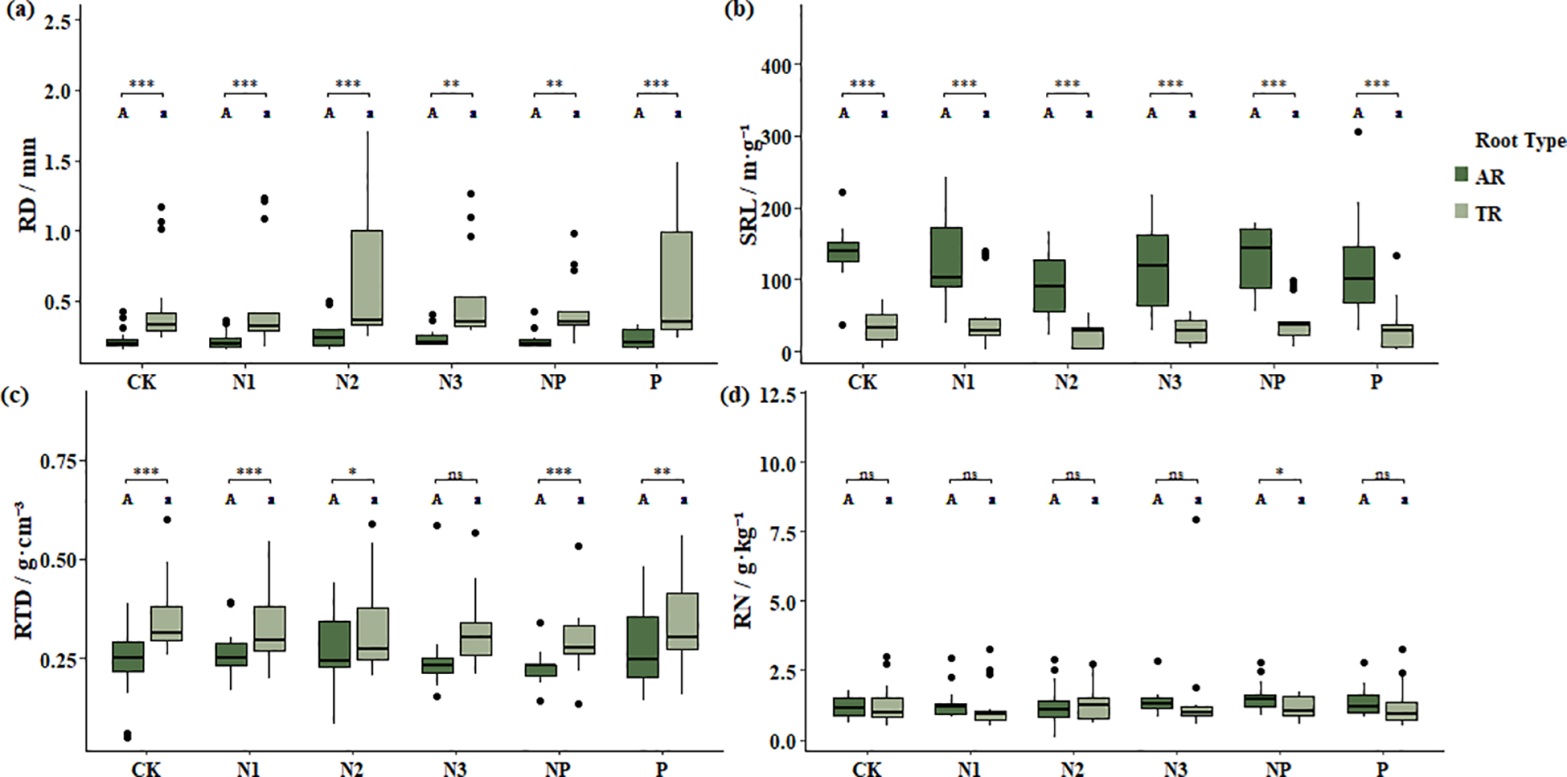
Comparison of functional traits of absorptive roots (AR) and transport roots (TR) under different treatments. **(a)** Root diameter (RD), **(b)** specific root length (SRL), **(c)** root tissue density (RTD), and **(d)** root nitrogen concentration (RN). Different uppercase letters indicate significant differences among absorptive roots, while different lowercase letters indicate significant differences among transport roots. *denotes significant differences between absorptive and transport roots within the same treatment; statistical significance is indicated as follows: ns (P > 0.05), *(P ≤ 0.05), **(P ≤ 0.01), and ***(P ≤  0.001).

Under nitrogen and phosphorus addition treatments, AR and TR exhibited consistent differentiation trends in morphological traits (**Figure 3**, **Supplementary Table S2**). TR showed significantly greater RD than AR across all treatments (P< 0.01), consistent with their roles in structural support and transport (**Figure 3a**). AR had significantly higher SRL than TR in all treatments (P< 0.001), indicating that AR maintained stronger foraging capacity for resource acquisition across nutrient conditions (**Figure 3b**). RTD was significantly higher in TR than AR in most treatments (P< 0.05), while no significant difference was detected under high-level nitrogen addition (N3) (P = 0.114), indicating that transport roots had denser tissue structures (**Figure 3c**). RN did not differ significantly between root types across treatments (P > 0.05), but AR showed a slightly higher RN than TR under combined nitrogen and phosphorus addition (NP) (1.52 ± 0.61 vs. 1.21 ± 0.49 g·kg^−1^, P< 0.05), implying enhanced nitrogen acquisition efficiency of AR when both nutrients were available (**Figure 3d**). These analyses were designed to test whether the functional contrasts between AR and TR remained consistent across different levels and combinations of nitrogen and phosphorus addition.

**Figure 4:**
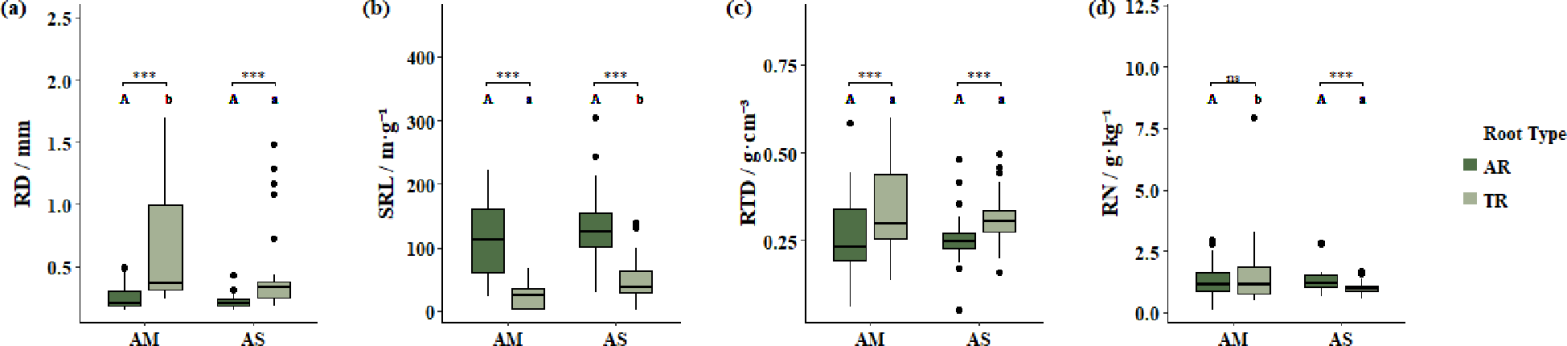
Comparison of functional traits between absorptive roots (AR) and transport roots (TR) across different grassland types. **(a)** Root diameter (RD), **(b)** specific root length (SRL), **(c)** root tissue density (RTD), and **(d)** root nitrogen concentration (RN). Different uppercase letters indicate significant differences among absorptive roots, while different lowercase letters indicate significant differences among transport roots. * denotes significant differences between absorptive roots and transport roots (ns P >0.05; ***P ≤ 0.001).

Comparisons across grassland types showed similar patterns (**Figure 4**, **Supplementary Table S2**). In both alpine meadow (AM) and alpine steppe (AS), AR had significantly smaller RD than TR (AM, 0.248 ± 0.092 mm vs. 0.616 ± 0.418 mm, P< 0.001; AS, 0.218 ± 0.055 mm vs. 0.423 ± 0.318 mm, P< 0.001). In AM, AR exhibited significantly higher SRL (131.6 ± 52.8 m·g^−1^) than TR (32.7 ± 21.5 m·g^−1^, P< 0.001), whereas TR showed significantly higher RTD than AR (0.338 ± 0.109 g·cm^−^3 vs. 0.246 ± 0.082 g·cm^−^3, P< 0.001). In AS, AR also had significantly higher SRL (118.3 ± 49.2 m·g^−1^) than TR (36.1 ± 25.4 m·g^−1^, P< 0.001), and TR exhibited greater RTD (0.342 ± 0.101 g·cm^−^3) compared with AR (0.263 ± 0.074 g·cm^−^3, P< 0.001). RN did not differ significantly between AR and TR in AM (1.29 ± 0.53 g·kg^−1^ vs. 1.21 ± 0.47 g·kg^−1^, P > 0.05), whereas in AS, AR had significantly higher RN than TR (1.38 ± 0.61 g·kg^−1^ vs. 1.07 ± 0.44 g·kg^−1^, P< 0.01).

### 3.3 Drivers for functional trait differences between absorptive roots and transport roots

Based on the three-factor linear mixed-effects model incorporating grassland type, nitrogen and phosphorus addition treatments, and plant functional groups, and assessed using Type-III ANOVA (**Table 1**), the differentiation in fine root traits between absorptive roots (AR) and transport roots (TR) exhibited trait-specific driving patterns. Specifically, differences in RD were significantly influenced only by plant functional groups (F = 20.17, df1 = 3, df2 ≈ 4.7, P = 0.004), whereas grassland type, nutrient addition, and their interactions were not significant. This indicates that species-specific characteristics mainly governed RD differentiation between AR and TR (**Supplementary Table S4**). Similarly, differences in SRL were also primarily driven by plant functional groups (F = 6.21, df1 = 3, df2 ≈ 4.6, P = 0.045), with no significant effects detected for other factors, indicating an inherent consistency in root elongation strategies across taxonomic groups. Differences in RN were not significantly affected by any main effect or interaction (P > 0.05), indicating that RN is relatively insensitive to grassland type and nutrient addition.

**Table 1:** Linear mixed-effects model analysis of drivers underlying functional trait differentiation between absorptive and transport roots.

Response^†^	Effect^‡^	df1	df2	F	P
RD	Type	1	16.7	4.03	0.061
	Treatment	5	4.3	0.37	0.849
	Family	3	4.7	20.17	0.004
	Type: Treatment	5	57.0	1.02	0.412
	Type: Family	2	11.4	0.53	0.601
	Treatment: Family	13	4.6	0.41	0.907
	Type: Treatment : Family	1	55.7	0.18	0.671
RN	Type	1	39.6	0.09	0.765
	Treatment	5	4.6	0.36	0.856
	Family	3	5.8	1.76	0.258
	Type: Treatment	5	57.3	1.67	0.157
	Type: Family	2	22.6	1.90	0.173
	Treatment: Family	13	5.2	0.97	0.561
	Type: Treatment : Family	1	56.9	1.61	0.210
SRL	Type	1	13.8	0.25	0.628
	Treatment	5	4.2	0.26	0.916
	Family	3	4.6	6.21	0.045
	Type: Treatment	5	56.7	0.61	0.694
	Type: Family	2	10.2	0.34	0.718
	Treatment: Family	13	4.5	0.30	0.960
	Type: Treatment : Family	1	55.6	0.11	0.738
RTD	Type	1	59.0	7.18	0.010
	Treatment	5	59.0	5.22	< 0.001
	Family	3	59.0	2.86	0.044
	Type: Treatment	5	59.0	0.26	0.933
	Type: Family	2	59.0	18.48	< 0.001
	Treatment: Family	13	59.0	4.61	< 0.001
	Type: Treatment : Family	1	59.0	0.07	0.796

^**†**^RD, root diameter; SRL, specific root length; RTD, root tissue density; RN, root nitrogen concentration.

^‡^Type: AM, AS; Treatment: CK, N1, N2, N3, NP, P; Family: *Poaceae*, *Fabaceae*, *Cyperaceae*, Others.

df1, degrees of freedom in the numerator; df2, degrees of freedom in the denominator.

In contrast, differentiation in RTD was significantly affected by grassland type (F = 7.18, P = 0.010), nitrogen and phosphorus addition (F = 5.22, P< 0.001), and plant functional groups (F = 2.86, P = 0.044). Furthermore, significant two-way interactions were observed between functional groups and both grassland type and nitrogen and phosphorus addition (P< 0.001). However, the interaction between grassland type and nutrient addition, as well as the three-way interaction, was not significant (P > 0.05). These findings indicate that RTD differentiation between AR and TR was jointly regulated by environmental conditions and species-specific traits, with heterogeneous responses among different plant functional groups.

### 3.4 Two-dimensional trait differences between absorptive roots and transport roots and trade-off among different traits

**Figure 5:**
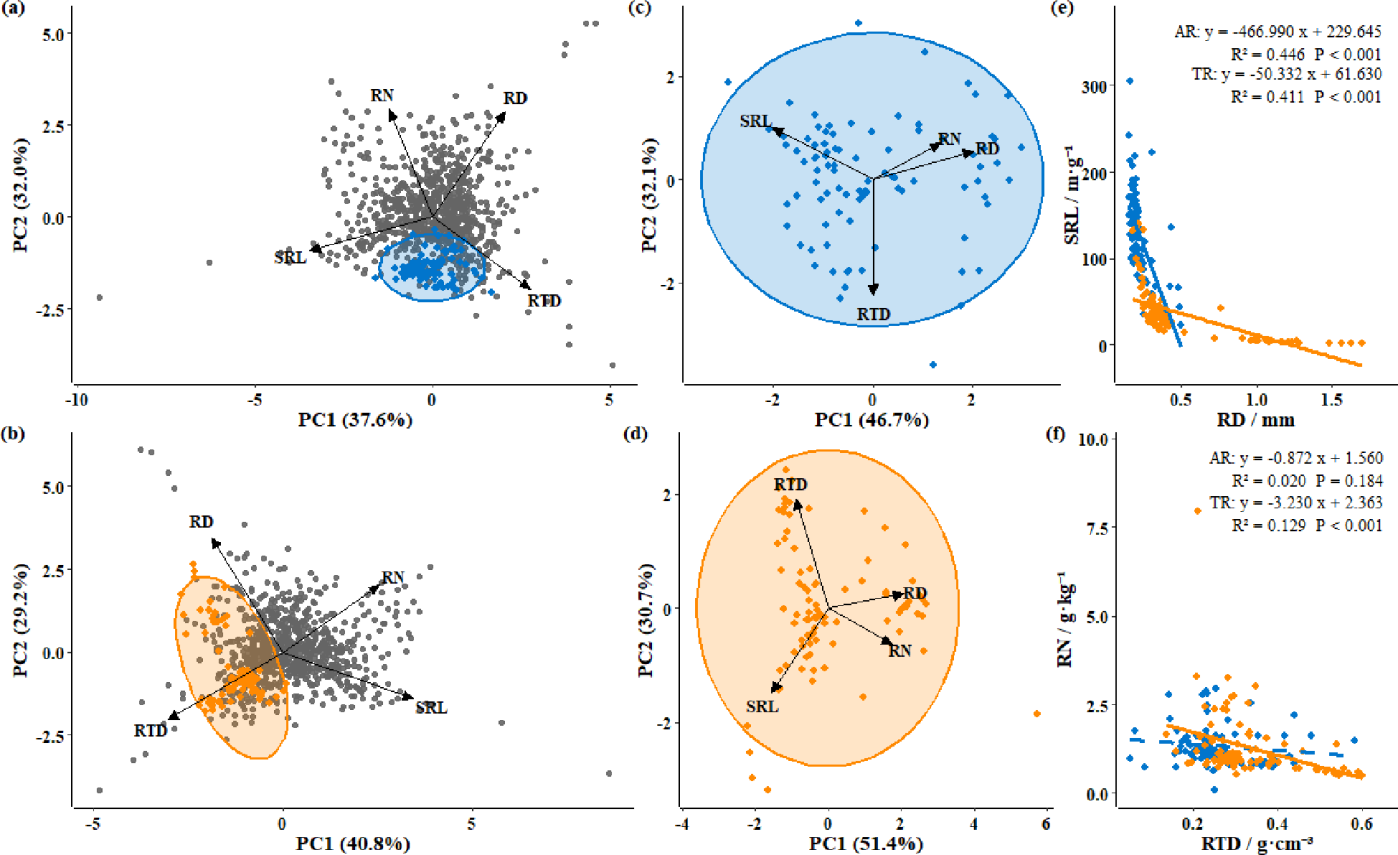
Principal component analysis (PCA) of functional traits in absorptive roots (AR) and transport roots (TR), with 95% bivariate confidence ellipses. Panel **(a)** shows the PCA of AR in a global context and the associated trade-off relationships among traits; Panel **(b)** shows the PCA of TR in a global context and the associated trade-off relationships among traits; Panel **(c)** displays the PCA of AR and the trade-off relationships among traits within this study; Panel **(d)** displays the PCA of TR and the trade-off relationships among traits within this study; Panel **(e)** depicts linear regression between root diameter (RD) and specific root length (SRL); Panel **(f)** depicts linear regression between root tissue density (RTD) and root nitrogen concentration (RN).

By projecting absorptive root (AR) and transport root (TR) samples from this study into the two-dimensional trait space constructed from global fine root databases (PC1 = acquisition–conservation axis; PC2 = independence–cooperation axis), we found that AR were mainly distributed in the lower region of the coordinate plane, indicating relatively higher RTD and SRL but lower RN compared with the global mean. TR were clustered in the lower-left region, suggesting higher RTD but lower SRL and RN relative to the global average (**Figures 5a, b**).

Principal component analysis revealed that the first two axes explained 78.8% of the total trait variation in AR (**Figure 5c**, **Supplementary Table S5**). PC1 explained 46.7% of the variation and was characterized by negative loadings of SRL and positive loadings of RD, RTD, and RN, reflecting differentiation along the ‘acquisition–conservation’ economic spectrum. PC2 explained 32.1% of the variation, with RTD loading negatively while RD, SRL, and RN loading positively, indicating a balance between tissue construction and nutrient content.

For TR, the first two axes explained 82.1% of the variation (**Figure 5d**, **Supplementary Table S5**). PC1 (51.4%) showed negative loadings for SRL and RTD and positive loadings for RD and RN, representing the primary gradient of the acquisition-conservation economic spectrum. PC2 (30.7%) exhibited negative loadings of RD and RTD and positive loadings of SRL and RN, indicating secondary balance between structural investment and nutrient acquisition.

Further analysis in the two-dimensional trait space exhibited that AR and TR still maintained dual trade-off patterns similar to those observed in the global coordinate system. Linear regression along the RD–SRL axis and the RTD–RN axis revealed significant differences between the two root types (P_RD–SRL< 0.001; P_RTD–RN = 0.043). Along the RD–SRL axis, both AR and TR showed significant negative correlations (AR: k = –466.99, R² = 0.446, P< 0.001; TR: k = –50.33, R² = 0.411, P< 0.001), confirming the consistency between fine root economic spectrum patterns on the QTP and global trait distributions (**Figure 5e**). Along the RTD-RN axis, only TR showed a significant negative correlation (AR: k = –0.872, R² = 0.020, P = 0.184; TR: k = –3.230, R² = 0.129, P< 0.001), indicating that the decoupling between tissue density and nitrogen content is not absolute but rather reflects a universal trade-off modulated by environmental conditions and functional types (**Figure 5f**).

In summary, the PCA and two-axis regression results jointly indicate that AR enhance resource acquisition efficiency by increasing SRL and RN. In contrast, TR improve improve transport capacity and mechanical support by increasing RD and RTD. These two root types thus represent ‘acquisitive’ and ‘conservative’ functional strategies, respectively, within the fine-root economic spectrum (RES), providing new empirical evidence for understanding the mechanisms underlying differentiation in below-ground economic strategies.

## 4 Discussions

### 4.1 Responses of functional traits of alpine grassland plants between absorptive roots and transport roots to nitrogen and phosphorus addition

Our results revealed significant differences between absorptive roots (AR) and transport roots (TR) in both morphological and chemical traits, highlighting clear functional differentiation within the fine root system. This differentiation was highly stable under nitrogen and phosphorus addition treatments across different types of grasslands. Specifically, AR exhibited higher specific root length (SRL) and lower root tissue density (RTD), reflecting a strategy optimized for rapid resource acquisition. In contrast, TR had lower SRL and higher RTD, indicative of a conservative strategy emphasizing mechanical support and structural stability. The consistent correspondence between morphology and function in both alpine meadow (AM) and alpine steppe (AS) suggests that functional differentiation of fine roots represents a typical ecological pattern (Atkinson et al., 2014), although its strength and expression can vary under extreme nutrient enrichment or between contrasting grassland types.

Under various nutrient addition treatments, this differentiation remained robust. Across sole nitrogen addition, phosphorus addition, and combined nitrogen-phosphorus addition treatments, the contrasting patterns of SRL and RTD between AR and TR persisted, demonstrating that plants maintain intrinsic functional differentiation even under changing nutritional conditions. This supports the view that fine root division of labour is a fundamental structural strategy for coping with environmental variability (Chen et al., 2024), rather than a transient response to nutrient availability. Only under high nitrogen addition (N3) did RTD differences between the two root types diminish, suggesting that excessive nitrogen input may weaken functional segregation within the root system, potentially affecting long-term stability. This weakening of RTD differentiation under the highest N addition (N3) is ecologically meaningful and may reflect a threshold response of fine-root systems to nutrient saturation. Under excessive nitrogen input, carbon allocation to structural tissues can decline due to lower construction costs and reduced demand for mechanical reinforcement, consistent with the concept of nitrogen saturation (Aber et al., 1998). Recent studies have reported that chronic nitrogen enrichment can reduce fine-root morphological differentiation and tissue density, indicating weakened structural investment and partial functional convergence among root orders (Zhu et al., 2021; Gao et al., 2023). Such convergence suggests that excessive nutrient supply may blur the functional boundary between absorptive and transport roots, leading to a loss of hierarchical specialization within fine-root systems. Therefore, the disappearance of RTD differences at N3 likely marks a physiological threshold beyond which nitrogen availability exceeds plant demand, constraining the functional division of labour and potentially undermining the structural stability of alpine root systems. Overall, nitrogen addition tended to enlarge the functional divergence between absorptive and transport roots by increasing SRL and RN in AR but having limited effects on TR, whereas phosphorus addition (alone or combined with N) exerted comparatively minor influence, indicating that P enrichment has a weaker effect on altering the intrinsic differentiation between AR and TR compared with nitrogen.

Across grassland types, the differentiation pattern was reinforced. In the drier AS, AR exhibited slightly lower SRL and slightly higher RTD than in AM, implying that enhanced structural reinforcement may facilitate reliable water and nutrient acquisition under water-limited conditions (Körner, 2003). This observation aligns with previously proposed structural adaptations of alpine plants, indicating that environmental context can shape root morphology in a directional and adaptive manner.

Overall, the functional divergence between AR and TR represents both an evolved structural feature and a stable ecological strategy for maintaining resource balance under variable environmental conditions. The persistence of this differentiation across nutrient treatments and grassland types indicates that hierarchical cooperation among roots constitutes a core mechanism enabling alpine plants to withstand harsh environments.

### 4.2 Stable functional differentiation between absorptive roots and transport roots of alpine grassland plants under nitrogen and phosphorus additions

The observed stability of functional differentiation suggests that the conservation of root architecture reflects long-term evolutionary adaptation (Ruili et al., 2018). Traits such as RD and SRL exhibited strong phylogenetic conservatism among plant functional groups, indicating that inherent taxonomic differences impose internal constraints on root functional strategies. Previous studies have shown that plant families differ inherently in root anatomical structure, and that this morphological conservatism provides the structural basis for adaptation to environmental stresses such as extreme cold and nutrient scarcity (Guo et al., 2008; Valverde-Barrantes et al., 2015).

Nitrogen and phosphorus addition treatments, as well as grassland types, appeared to influence RTD differences between AR and TR, as suggested by the patterns observed in **Figure 3** and **Figure 4**. In particular, nitrogen addition showed a concentration-dependent trend, where RTD differences between AR and TR gradually decreased with increasing N input. This may be associated with accelerated root growth under high nitrogen conditions, an increased proportion of thin-walled tissues, and altered carbon allocation strategies within the root system (Freschet et al., 2010; De Vries and Bardgett, 2016; Zhu et al., 2021). Although overall functional differentiation remained stable, excessive nitrogen input could weaken structural and defensive differences between AR and TR, potentially affecting long-term resource utilization and root system stability (Aber et al., 1998; Treseder, 2008).

High RTD is typically associated with lower tissue water content and reduced decomposability per unit volume, coupled with greater carbon investment and longer lifespan (Ryser, 1996; Pregitzer et al., 1997), reflecting a conservative functional strategy. In the nutrient-poor, cold alpine ecosystems of the QTP, maintaining high RTD can reduce tissue freezing and mechanical damage, thereby enhancing plant survival (Körner, 2003; Reich, 2014). Collectively, the functional divergence between AR and TR is shaped by both genetic evolution and environmental regulation: genetics establishes the fundamental pattern, while environmental conditions shapes fine-tune trait expression. RTD serves as a key mediating trait, constrained by phylogeny yet responsive to environmental stress, forming the core mechanism underpinning fine root functional differentiation.

Although nitrogen and phosphorus addition did not fundamentally alter the overall functional differentiation between AR and TR, they may reflect indirect influences of soil nutrient availability and potential mycorrhizal associations on the relationship between RTD and root nitrogen concentration (RN) (Averill and Hawkes, 2016; Soudzilovskaia et al., 2019). These patterns are consistent with possible variations in mycorrhizal association and root turnover that jointly influence root construction and nutrient acquisition strategies (McCormack et al., 2015; Bergmann et al., 2020). However, anatomical traits and mycorrhizal colonization were not directly measured in this study; therefore, related interpretations should be regarded as conceptual and inferential rather than demonstrated. Notably, under combined nitrogen–phosphorus addition, RN in absorptive roots showed a slight but non-significant increase, suggesting only a marginal trend rather than a clear functional shift in nutrient uptake. Distinct mycorrhizal symbioses are known to influence root morphology and can regulate both RTD and nutrient acquisition efficiency (Brundrett, 2009). Overall, the minor RN differences between AR and TR observed here are more plausibly controlled by physiological or symbiotic factors than by direct environmental effects.

In summary, functional divergence between absorptive and transport roots reflects the interplay of genetic evolution and environmental regulation. RTD acts as a central mediating trait, simultaneously phylogenetically constrained and environmentally responsive, and underpins the hierarchical functional differentiation within fine root systems.

### 4.3 Similarity and difference of root economics spectrum in alpine grassland plants on QTP with global patterns

Within the global framework of the root economics spectrum (RES), AR and TR of alpine plants on the QTP maintain a balance along the SRL-RD and RTD-RN axes, consistent with the widely recognized dual-dimensional patterns of acquisition-conservation and independence-cooperation strategies. However, this universal pattern is superimposed by the region’s unique characteristics as the ‘Third Pole’ of the world.

Globally, AR with high SRL and low Root Tissue Density (RTD) reflect a low-cost, rapid resource acquisition strategy, whereas TR with high RTD and low SRL represent a conservative, long-lived structural strategy (Weemstra et al., 2016; Freschet et al., 2021). In our study, AR and TR on the QTP formed two distinct and stable clusters within this framework, indicating that fine root division of labour is a widespread ecological rule across ecosystems (Prieto et al., 2015; Freschet et al., 2021). More importantly, under the cold and nutrient-poor conditions of the QTP, plants tend to shift toward the structural reinforcement end of the trait space, characterised by elevated RTD. This suggests that plants must incur higher construction costs to withstand freezing–thawing cycles and water stress, reflecting adaptive trade-offs in alpine ecosystems.

Moreover, alpine plants on the QTP exhibited coordinated adjustments in both structural and metabolic dimensions. Binter et al. (2025) highlighted that root traits in cold environments are primarily determined by anatomical construction rather than classical morphological indicators. Consistently, RD and RTD showed generally independent but directionally aligned variation across species, without a clear negative trade-off pattern. This suggests that plants in alpine environments may enhance mechanical stability and hydraulic safety mainly through tissue densification, rather than by increasing root diameter alone. This pattern contrasts with the ‘cortex–stele decoupling’ observed in other contexts Cao et al. (2025), indicating stronger anatomical constraints in cold alpine ecosystems.

Additionally, a negative correlation between RTD and RN was observed, which was significant in transport roots but weaker in absorptive roots. This pattern still indicates that the trade-off between nutrient metabolism and structural reinforcement tends to intensify under extreme environmental conditions (Reich, 2014). In line with the dual-axis economic spectrum proposed by Kong et al. (2014) and Zhang et al. (2024), fine roots on the Plateau develop a distinctive economic strategy characterised by relatively high RTD accompanied by decreasing SRL from absorptive to transport roots, reflecting a structural and metabolic trade-off along the root branching hierarchy. This pattern reflects both the ecological adaptation of alpine roots and the evolutionary constraints imposed by environmental extremes.It should be emphasized that the ‘independence–cooperation’ axis discussed here is conceptually inferred from global literature on the root economics spectrum (Kong et al., 2019; Bergmann et al., 2020). Our study did not directly measure root anatomical traits or mycorrhizal colonization. Therefore, any statements about fungal collaboration, cortical allometry, or cooperative strategies are hypotheses for future work rather than tested mechanisms in this study. Nevertheless, the observed RTD and RN patterns provide a valuable empirical context for linking alpine root trait variation to global frameworks.

The PCA results in this study partially support the root economic space hypothesis, with the first axis corresponding to the acquisition–conservation trade-off driven by SRL, RD, and RN, while the second axis was dominated by RTD. This differs from global datasets, where RTD typically loads inversely on the first axis. The discrepancy likely arises from the unique selective pressures of alpine ecosystems, including low temperature, short growing seasons, and nutrient limitation, under which tissue density may vary independently from absorptive efficiency. This reflects an adaptive strategy that balances mechanical stability and hydraulic safety rather than strictly following the global acquisitive–conservative continuum.

In summary, the RES of plants on the QTP retains the globally recognised patterns while simultaneously establishing a unique functional balance under extreme environmental pressures through structural reinforcement and metabolic coordination. Alpine plants on the QTP allocate more resources to structural construction to tolerate freezing, thawing, and water stress, highlighting a specialised fine root strategy that reflects adaptive adjustment to harsh alpine conditions.

## 5 Conclusions

Our study suggests a clear and stable functional differentiation between absorptive roots (AR) and transport roots (TR) in alpine meadow and alpine steppe ecosystems of the QTP under nitrogen and phosphorus addition. AR adopt a resource-acquisitive strategy with high specific root length and elevated nitrogen concentration, whereas TR exhibit a conservative, structurally supportive strategy characterized by higher root tissue density. This functional division of labour remains robust across nutrient treatments and grassland types, though RTD differentiation is particularly sensitive to environmental factors, notably nitrogen enrichment. Phosphorus addition, either alone or combined with nitrogen, exerted comparatively limited influence on root functional differentiation, suggesting that nitrogen plays a more dominant role in modulating fine-root trait expression on the Qinghai–Tibet Plateau. These findings reveal how alpine plants balance resource acquisition and structural investment, reflecting adaptive strategies to the cold, nutrient-limited conditions of the QTP. Integrating anatomical, physiological, and microbial perspectives in future studies will further elucidate the mechanistic basis of fine root functional differentiation and its role in sustaining ecosystem functioning under global change. In addition, although phylogenetic relationships were not considered in this study due to limited taxonomic coverage, incorporating phylogenetic signal analyses in future research would help clarify whether the observed trait differentiation between absorptive and transport roots reflects primarily environmental adaptation or phylogenetic constraints.

## Data Availability

The original contributions presented in the study are included in the article/**Supplementary Material**. Further inquiries can be directed to the corresponding authors.
