# Supplementary material for: Functional differentiation and adaptive responses of absorptive and transport roots in alpine grassland plants under nitrogen and phosphorus addition
Source: Front Plant Sci. 2026 Jan 15;16:1747072. doi: 10.3389/fpls.2025.1747072 (PMC12852885; doi:10.3389/fpls.2025.1747072)
Supplement: Supplementary file 1 [file Table1.docx]

Supplementary Material

## Descriptive statistics of functional traits for absorptive roots and transport roots

| **Group** | **Metric^†^** | **Root Order** | **Mean** | **SD** | **CV** | **N** | **P** |
| --- | --- | --- | --- | --- | --- | --- | --- |
| All | RD | Absorptive roots | 0.236 | 0.080 | 0.340 | 90 | < 0.001 |
|  |  | Transport roots | 0.539 | 0.391 | 0.725 |  |  |
|  | RN | Absorptive roots | 1.334 | 0.551 | 0.413 | 90 | 0.640 |
|  |  | Transport roots | 1.290 | 0.964 | 0.747 |  |  |
|  | RTD | Absorptive roots | 0.259 | 0.089 | 0.344 | 90 | < 0.001 |
|  |  | Transport roots | 0.332 | 0.107 | 0.323 |  |  |
|  | SRL | Absorptive roots | 119.575 | 56.089 | 0.469 | 90 | < 0.001 |
|  |  | Transport roots | 34.497 | 30.652 | 0.889 |  |  |

† RD, root diameter; SRL, specific root length; RTD, root tissue density; RN, root nitrogen concentration.

CV, Coefficient of Variation; N, Sample Size.

## Descriptive statistics of functional traits for absorptive and transport roots under different nitrogen and phosphorus addition treatments and grassland types

| **Group** | **Metric^†^** | **Root Order** | **Mean** | **SD** | **CV** | **N** | **P** |
| --- | --- | --- | --- | --- | --- | --- | --- |
| CK | RD | Absorptive roots | 0.224 | 0.082 | 0.367 | 16 | 0.001 |
|  |  | Transport roots | 0.463 | 0.316 | 0.683 |  |  |
|  | RN | Absorptive roots | 1.191 | 0.392 | 0.329 | 16 | 0.621 |
|  |  | Transport roots | 1.275 | 0.725 | 0.569 |  |  |
|  | RTD | Absorptive roots | 0.242 | 0.094 | 0.389 | 16 | < 0.001 |
|  |  | Transport roots | 0.348 | 0.090 | 0.258 |  |  |
|  | SRL | Absorptive roots | 137.841 | 37.459 | 0.272 | 16 | < 0.001 |
|  |  | Transport roots | 34.304 | 22.182 | 0.647 |  |  |
| N1 | RD | Absorptive roots | 0.217 | 0.065 | 0.297 | 16 | 0.004 |
|  |  | Transport roots | 0.468 | 0.359 | 0.767 |  |  |
|  | RN | Absorptive roots | 1.311 | 0.565 | 0.431 | 16 | 0.221 |
|  |  | Transport roots | 1.175 | 0.808 | 0.688 |  |  |
|  | RTD | Absorptive roots | 0.271 | 0.067 | 0.248 | 16 | < 0.001 |
|  |  | Transport roots | 0.343 | 0.112 | 0.327 |  |  |
|  | SRL | Absorptive roots | 125.001 | 61.770 | 0.494 | 16 | < 0.001 |
|  |  | Transport roots | 46.215 | 46.635 | 1.009 |  |  |
| N2 | RD | Absorptive roots | 0.273 | 0.115 | 0.422 | 16 | 0.001 |
|  |  | Transport roots | 0.701 | 0.534 | 0.761 |  |  |
|  | RN | Absorptive roots | 1.250 | 0.731 | 0.584 | 16 | 0.504 |
|  |  | Transport roots | 1.348 | 0.677 | 0.502 |  |  |
|  | RTD | Absorptive roots | 0.273 | 0.098 | 0.360 | 16 | 0.033 |
|  |  | Transport roots | 0.330 | 0.126 | 0.381 |  |  |
|  | SRL | Absorptive roots | 90.837 | 43.613 | 0.480 | 16 | < 0.001 |
|  |  | Transport roots | 23.079 | 17.671 | 0.766 |  |  |
| N3 | RD | Absorptive roots | 0.243 | 0.072 | 0.296 | 12 | 0.006 |
|  |  | Transport roots | 0.530 | 0.357 | 0.673 |  |  |
|  | RN | Absorptive roots | 1.388 | 0.510 | 0.367 | 12 | 0.726 |
|  |  | Transport roots | 1.597 | 2.031 | 1.272 |  |  |
|  | RTD | Absorptive roots | 0.255 | 0.110 | 0.431 | 12 | 0.114 |
|  |  | Transport roots | 0.321 | 0.100 | 0.313 |  |  |
|  | SRL | Absorptive roots | 116.634 | 63.031 | 0.540 | 12 | < 0.001 |
|  |  | Transport roots | 29.331 | 18.947 | 0.646 |  |  |
| NP | RD | Absorptive roots | 0.227 | 0.069 | 0.305 | 13 | 0.001 |
|  |  | Transport roots | 0.441 | 0.236 | 0.536 |  |  |
|  | RN | Absorptive roots | 1.590 | 0.549 | 0.345 | 13 | 0.018 |
|  |  | Transport roots | 1.163 | 0.389 | 0.334 |  |  |
|  | RTD | Absorptive roots | 0.227 | 0.046 | 0.201 | 13 | < 0.001 |
|  |  | Transport roots | 0.296 | 0.090 | 0.305 |  |  |
|  | SRL | Absorptive roots | 129.888 | 45.222 | 0.348 | 13 | < 0.001 |
|  |  | Transport roots | 41.985 | 31.174 | 0.743 |  |  |
| P | RD | Absorptive roots | 0.231 | 0.063 | 0.272 | 17 | < 0.001 |
|  |  | Transport roots | 0.607 | 0.433 | 0.714 |  |  |
|  | RN | Absorptive roots | 1.334 | 0.505 | 0.379 | 17 | 0.314 |
|  |  | Transport roots | 1.241 | 0.761 | 0.613 |  |  |
|  | RTD | Absorptive roots | 0.280 | 0.105 | 0.375 | 17 | 0.003 |
|  |  | Transport roots | 0.344 | 0.123 | 0.357 |  |  |
|  | SRL | Absorptive roots | 118.514 | 72.633 | 0.613 | 17 | < 0.001 |
|  |  | Transport roots | 32.316 | 33.454 | 1.035 |  |  |
| AM | RD | Absorptive roots | 0.248 | 0.092 | 0.371 | 54 | < 0.001 |
|  |  | Transport roots | 0.616 | 0.418 | 0.677 |  |  |
|  | RN | Absorptive roots | 1.347 | 0.614 | 0.456 | 54 | 0.348 |
|  |  | Transport roots | 1.479 | 1.193 | 0.806 |  |  |
|  | RTD | Absorptive roots | 0.264 | 0.101 | 0.384 | 54 | < 0.001 |
|  |  | Transport roots | 0.346 | 0.125 | 0.362 |  |  |
|  | SRL | Absorptive roots | 112.455 | 55.285 | 0.492 | 54 | < 0.001 |
|  |  | Transport roots | 24.300 | 17.994 | 0.741 |  |  |
| AS | RD | Absorptive roots | 0.218 | 0.055 | 0.253 | 36 | < 0.001 |
|  |  | Transport roots | 0.423 | 0.318 | 0.751 |  |  |
|  | RN | Absorptive roots | 1.314 | 0.447 | 0.340 | 36 | < 0.001 |
|  |  | Transport roots | 1.007 | 0.265 | 0.263 |  |  |
|  | RTD | Absorptive roots | 0.253 | 0.068 | 0.270 | 36 | < 0.001 |
|  |  | Transport roots | 0.311 | 0.069 | 0.222 |  |  |
|  | SRL | Absorptive roots | 130.255 | 56.356 | 0.433 | 36 | < 0.001 |
|  |  | Transport roots | 49.793 | 38.700 | 0.777 |  |  |

† CK, control group; N1, low nitrogen addition; N2, medium nitrogen addition; N3, high nitrogen addition; P, phosphorus addition; NP, combined nitrogen-phosphorus addition; AM, Alpine Meadow; AS, Alpine Steppe.

CV, Coefficient of Variation; N, Sample Size.

## Nutrient addition treatments and corresponding application rates in alpine meadow and alpine steppe

| **Treatment†** | **Description** | **N Application rate**  **(kg·ha^-1^·yr^-1^)** | **P Application rate**  **(kg·ha^-1^·yr^-1^)** | **Nutrient source** | **Reference** |
| --- | --- | --- | --- | --- | --- |
| CK | No nutrient addition | 0 | 0 |  |  |
| N1 | Low N addition | 8 | 0 | NH₄NO₃ | Shen et al. (2022) |
| N2 | Medium N addition | 72 | 0 | NH₄NO₃ | Xiao et al. (2025) |
| N3 | High N addition | 216 | 0 | NH₄NO₃ | Xiao et al. (2025) |
| P | P addition | 0 | 35 | Ca(H₂PO₄)₂ | Shen et al. (2022) |
| NP | combined N-P addition | 72 | 35 | NH₄NO₃ + Ca(H₂PO₄)₂ | Xiao et al. (2025) |

† CK, control group; N1, low nitrogen addition; N2, medium nitrogen addition; N3, high nitrogen addition; P, phosphorus addition; NP, combined nitrogen-phosphorus addition; AM, Alpine Meadow; AS, Alpine Steppe.

## Regression statistics for pairwise relationships among key root traits

| **Root Type†** | **Trait Pair** | **Equation** | **N** | **R²** | **P** | **k** | **b** |
| --- | --- | --- | --- | --- | --- | --- | --- |
| AR | RD–SRL | SRL = −0.87 × RD + 3.25 | 60 | 0.68 | < 0.001 | −0.87 | 3.25 |
| TR | RD–SRL | SRL = −0.45 × RD + 1.90 | 58 | 0.51 | 0.002 | −0.45 | 1.90 |
| AR | RTD–RN | RN = −0.32 × RTD + 2.47 | 60 | 0.56 | 0.004 | −0.32 | 2.47 |
| TR | RTD–RN | RN = −0.27 × RTD + 2.02 | 58 | 0.42 | 0.013 | −0.27 | 2.02 |

† AR, absorptive roots; TR, transport roots.

N, Sample Size; R², Coefficient of Determination; k, Slope; b, Intercept.

## Summary of Principal Component Analysis (PCA) of different fine root traits

| **Analysis** | **PC^†^** | **Eigenvalue^‡^** | **Variance explained (%)^‡^** | **Trait** | **PC1 loading** | **PC2 loading** |
| --- | --- | --- | --- | --- | --- | --- |
| AR | PC1 | 1.8683 | 46.71 | RD | 0.8734 | 0.2397 |
|  |  |  |  | SRL | −0.8699 | 0.4362 |
|  |  |  |  | RTD | 0.0097 | −0.9712 |
|  |  |  |  | RN | 0.5905 | 0.3040 |
|  | PC2 | 1.2833 | 32.08 | — | — | — |
| TR | PC1 | 2.0565 | 51.41 | RD | 0.9133 | 0.1093 |
|  |  |  |  | SRL | −0.6865 | −0.6544 |
|  |  |  |  | RTD | −0.3942 | 0.8410 |
|  |  |  |  | RN | 0.7718 | −0.2819 |
|  | PC2 | 1.2269 | 30.67 | — | — | — |
| Global | PC1 | 1.5668 | 39.17 | RD | −0.4355 | 0.7568 |
|  |  |  |  | SRL | 0.7896 | −0.3425 |
|  |  |  |  | RTD | −0.7063 | −0.4402 |
|  |  |  |  | RN | 0.5048 | 0.5727 |
|  | PC2 | 1.2118 | 30.30 | — | — | — |
| AR + Global | PC1 | 1.5045 | 37.61 | RD | 0.4971 | 0.6992 |
|  |  |  |  | SRL | −0.8466 | −0.2244 |
|  |  |  |  | RTD | 0.6713 | −0.4817 |
|  |  |  |  | RN | −0.3000 | 0.7138 |
|  | PC2 | 1.2809 | 32.02 | — | — | — |
| TR + Global | PC1 | 1.6305 | 40.76 | RD | −0.4273 | 0.7948 |
|  |  |  |  | SRL | 0.7890 | −0.3227 |
|  |  |  |  | RTD | −0.6942 | −0.4645 |
|  |  |  |  | RN | 0.5861 | 0.4637 |
|  | PC2 | 1.1666 | 29.16 | — | — | — |

**† PC represents the principal component axis.**

‡ **Eigenvalue and Variance explained (%) show the amount and proportion of total variance explained by each axis.**

**AR, absorptive roots; TR, transport roots; Global, GRooT database.**
